# Physicians’ professional performance: an occupational health psychology perspective

**DOI:** 10.1007/s40037-017-0382-9

**Published:** 2017-10-24

**Authors:** Renée A. Scheepers

**Affiliations:** Professional Performance Research Group, Center for Evidence-Based Education, Academic Medical Center, University of Amsterdam, Amsterdam, The Netherlands

**Keywords:** Professional performance, Clinical teaching practice, Residency training, Teaching performance, Patient care experience, Patient care quality, Work engagement, Job resources, Personality traits

## Abstract

**Introduction:**

Physician work engagement is considered to benefit physicians’ professional performance in clinical teaching practice. Following an occupational health psychology perspective, this PhD report presents research on how physicians’ professional performance in both doctor and teacher roles can be facilitated by work engagement and how work engagement is facilitated by job resources and personality traits.

**Methods:**

First, we conducted a systematic review on the impact of physician work engagement and related constructs (e. g. job satisfaction) on physicians’ performance in patient care. We additionally investigated physician work engagement and job resources in relation to patient care experience with physicians’ performance at ten outpatient clinics covering two hospitals. In a following multicentre survey involving 61 residency training programs of 18 hospitals, we studied associations between physician work engagement and personality traits with resident evaluations of physicians’ teaching performance.

**Results:**

The findings showed that physician work engagement was associated with fewer reported medical errors and that job satisfaction was associated with better communication and patient satisfaction. Autonomy and learning opportunities were positively associated with physician work engagement. Work engagement was positively associated with teaching performance. In addition, physician work engagement was most likely supported by personality trait conscientiousness (e. g. responsibility).

**Conclusion:**

Given the reported associations of physician work engagement with aspects of their professional performance, hospitals could support physician work engagement in service of optimal performance in residency training and patient care. This could be facilitated by worker health surveillance, peer support or promoting job crafting at the individual or team level.

## Introduction

For residents to become experts in specialized patient care, they are required to be trained by physicians showing professional performance in both doctor and teacher roles. Physicians’ professional performance can be defined as all the actions or processes in performing work tasks, whilst adhering to the values and behaviours of the medical profession [1]. In both doctor and teacher roles standards on professional performance are continuously updated following new scientific insights, policy developments and evolving societal expectations. Physicians are faced with a progressing complexity of clinical teaching practice, high workloads and growing bureaucracy. These developments have increasingly led the medical community to express concerns about physicians’ stress and well-being in their work. Ultimately, physician well-being is considered to benefit professional performance in patient care and residency training. This PhD report presents an occupational health psychology perspective on physician well-being and professional performance in both doctor and teacher roles. In occupational health psychology, well-being has been widely conceptualized as work engagement [2]. Work engagement is defined as a fulfilling, active-motivational state of positive work-related well-being. Work-engaged physicians are less likely to suffer from burnout and are dedicated and energetic in their work. Specific work characteristics, i. e. job resources, have been shown to foster work engagement [3]. In addition, also individual characteristics, such as personality traits, can facilitate professionals in their work engagement [4]. In this thesis we studied whether and which job resources as well as personality traits may facilitate physician work engagement, and how physician work engagement is associated with professional performance in doctor and teacher roles.

## Methods

Our first study included a systematic review on physician well-being in relation to professional performance in their doctor role – i. e. the quality of patient care provided by individual physicians [5]. The review was guided by the Preferred Reporting Items for Systematic Reviews and Meta-Analyses [6]. For physician well-being, we studied work engagement as well as related constructs from occupational health psychology (e. g. job satisfaction) to provide a comprehensive overview on the topic. We developed an extensive search strategy for PubMed, Embase and Psychinfo, which included both index and free terms on the subjects of well-being (e. g. work engagement), patient care quality (e. g. patient satisfaction) and physicians (e. g. doctors). The eligibility of the articles was assessed by two reviewers and study quality was determined using the Medical Education Research Study Quality Instrument (MERSQI) [7].

In the second study we zoomed in on the association between physician work engagement and their professional performance based on patient care experience [8]. In addition, we identified which job resources were supportive for work engagement of physicians. Patient care experience was measured at ten outpatient clinics covering two academic medical centres, using nine validated items on a 5-point scale from the Consumer Quality Index [9]. Physicians reported their work engagement on a 7-point scale using the validated 9‑item Utrecht Work Engagement Scale (UWES-9) [10] and their job resources on a 5-point scale using the validated Questionnaire on the Evaluation of Work (QEEW) [11]. Job resources included autonomy, opportunities to learn and develop, participation in decision making and colleague support. We conducted linear regression analysis and linear mixed models to account for clustering of patients within physicians.

The third study investigated the association between work engagement and physicians’ professional performance in their teacher role [12]. Physicians’ performance in their teacher role was determined by the validated System for Evaluation of Teaching Qualities (SETQ) [13], measuring resident-evaluated teaching performance and role model status of physician supervisors on a 5-point scale. We invited 815 residents and 819 physicians of 61 residency training programs and 18 medical centres for this study. Physicians reported their work engagement in both doctor and teacher roles, using the UWES-9 [10]. We performed a t-test to assess whether physicians showed more engagement in their doctor or teacher work and generalized estimating equations on associations between (doctor versus teacher) work engagement and teaching performance as well as role model status, accounting for the multilevel setting of the study.

In the fourth study, the same sample was used to study how physicians’ work engagement in doctor and teacher roles was facilitated by personality traits [14]. To that end, physician supervisors reported personality traits using the well-validated 10-item Big Five Inventory [15], measuring personality traits in five domains: extraversion, emotional stability, conscientiousness, agreeableness and openness. We conducted structural equation modelling analysis to study associations between personality traits, work engagement and teaching performance.

## Results

The first study yielded 5,944 unique hits, of which 18 were eligible for our systematic review. Most of these studies employed an observational design and were of average quality. The studies indicated that physician well-being in terms of job satisfaction was positively associated with interpersonal aspects of performance (such as communication towards patients), patient satisfaction and patient adherence to physicians’ treatment recommendations. Furthermore, physician work engagement specifically was associated with fewer self-reported medical errors.

The second study included 4,573 patients (47%) reporting patient care experience, resulting in an average of 22.3 patient evaluations per physician. In total, 185 physicians (78%) participated. Work engagement was not associated with patient care experience (*B* = 0.01, 95% CI −0.02–0.03, *p* = 0.669). Autonomy (*B* = 0.31, 95% CI 0.10 to 0.51, *p* = 0.004) and opportunities to learn and develop (*B* = 0.28, 95% CI 0.05 to 0.52, *p* = 0.019) were positively associated with work engagement.

In the third study, 549 residents (68%) and 636 physicians (78%) participated, leading to an average of 5.43 resident evaluations per physician. Physicians were on average more engaged as doctors than as teachers (mean difference 0.95, 95% CI 0.86–1.04, *p* < 0.001). However, higher levels of supervisors’ engagement in their teacher work were associated with better teaching performance (*B* = 0.11; 95% CI 0.08–0.14, *p* < 0.001) while higher levels of doctor work engagement were not associated with better teaching performance (*B* = −0.03, 95% CI −0.07–0.01, *p* = 0.186). Ultimately, supervisors with higher levels of teaching performance were evaluated as more positive role models by residents (*B* = 1.08; 95% CI 0.10–1.18, *p* < 0.001).

The fourth study showed that teacher work engagement was positively affected by agreeableness (*B* = 0.25, *SE* = 0.13, *p* = 0.047). Emotional stability was positively associated with doctor work engagement specifically (*B* = 0.23, *SE* = 0.08, *p* = 0.004). Conscientiousness was positively associated with both doctor work engagement (*B* = 2.36, *SE* = 0.60, *p* < 0.001;) and teacher work engagement (*B* = 1.81, *SE* = 0.41, *p* < 0.001).

## Discussion

The main findings of this thesis show that physician well-being is associated with aspects of their professional performance. Physician well-being in terms of job satisfaction showed to positively affect adequate communication towards patients and patient satisfaction. Physicians who were specifically work-engaged reported fewer medical errors and work-engaged teachers were evaluated as better performing supervisors. The thesis findings align with related research on physician well-being; a recent meta-analysis reported that health care professional burnout is associated with lower levels of patient care quality and safety [16]. In this thesis, we specifically focused on work engagement as an indicator of positive well-being. Physicians experiencing more work engagement face less stress, and have more energy and mental resources to direct full attention to the needs of patients or residents [17]. Residents did indeed recognize better performing supervisors in those who were highly engaged. This specifically applied to supervisors engaged in teaching. However, as reported by the present and previous research, physicians are on average more engaged for patient care than for teaching [18]. One of the possible clarifications for these different work engagement levels may follow from tensions in patient care versus supervisory responsibilities in daily practice. Research shows that in dealing with this twofold responsibility, physicians may come to prioritize safe patient care over trainee learning [19]. In addition, physicians perceive to be less extensively prepared for teaching responsibilities than for medical competencies [20]. To that regard, more resources and evidence-based faculty development programs for physicians in clinical teaching practice may contribute to physicians’ engagement for teaching, subsequently beneficial for their performance.

Since autonomy and learning opportunities were shown to benefit work engagement, these working conditions should be optimized. In addition, personality traits made a difference for how engaged physicians were in their doctor and teacher roles, as emotional stability specifically facilitated physicians’ work engagement as doctors and agreeable physicians were more likely to be engaged teachers. Therefore, individualized support for physicians’ work engagement in patient care and resident supervision could be considered, which could be embedded by training physicians in job crafting, involving proactive behaviours in purposefully enhancing energizing aspects of work and alleviating exhausting aspects of work in clinical teaching practice [21].

In general, the results of this thesis were based on extensively validated instruments and could be further enhanced by also conducting qualitative studies on this topic. For organizations to further support physician work engagement or well-being, implementing worker health surveillance or peer support could be considered [22]. Worker health surveillance is focused at improving or maintaining well-being through treating or preventing work-related health complaints [22] and peer support has shown to be auxiliary for physicians in coping with the stresses of clinical teaching practice [23]. Ultimately, the well-being of both patients and residents is served by the exposure to physician role models who are experts in maintaining and improving their own well-being.

### A piece of advice

A PhD trajectory offers much autonomy to professionally develop yourself as a researcher. My advice would be to use that autonomy and pro-actively craft your PhD training according to your personal and professional ambitions. Discuss these ambitions with your supervisors and invite experts to collaborate with you in research.

### Thesis information

The PhD defence took place at the University of Amsterdam in the Netherlands on 26 February 2016. The thesis was conducted in the Professional Performance research group of the Academic Medical Center in Amsterdam and was supervised by the promotors Prof. Kiki Lombarts, Prof. Maas Jan Heineman (Academic Medical Center, University of Amsterdam) and co-promotor Prof. Onyebuchi Arah (University of California, Los Angeles). The research reported in this thesis was financially supported by the Dutch Ministry of Health, Welfare and Sports and by the Royal Dutch Association of Advancement in Medicine (KNMG).
